# Effect of graphene / metal nanocomposites on the key genes involved in rosmarinic acid biosynthesis pathway and its accumulation in *Melissa officinalis*

**DOI:** 10.1186/s12870-021-03052-z

**Published:** 2021-06-05

**Authors:** Roya Karimi Soraki, Mahyar Gerami, Moazzameh Ramezani

**Affiliations:** 1 Medicinal Plants Department, Sana Institute of Higher Education, Sari, Iran; 2 Plant Physiology Department, Faculty of Sana Institute of Higher Education, Sari, Iran; 3 Urmia University, Urmia, Iran

**Keywords:** Silver nanoparticle, Graphene, Nanocomposite, *Melissa officinalis*, Rosmarinic acid

## Abstract

**Background:**

Recently, numerous investigations have been done to study graphene and silver nanoparticle in the fields of agriculture and medicine*.* In the present study, the green synthesis of nanoparticles with two concentrations (0, 40, 60 mM) and their effect on the molecular and biochemical biosynthesis pathway of rosmarinic acid in a new method, low cost, and safe for the environment has been investigated. The transcript levels of key genes in the rosmarinic acid biosynthesis pathway (Tyrosine aminotransferase, rosmarinic acid synthase, and phenylalanine-ammonia lyase) were studied using real-time quantitative polymerase chain reaction. Then, the rosmarinic acid content was evaluated using HPLC.

**Results:**

The results showed that a concentration-dependent manner was observed in treated plants. At the biochemical level, the use of nanocomposites at concentration of 40 mM showed higher soluble carbohydrate (37%), flavonoids (21%), total phenol (35%) as well as total protein (47%) compared to the control plants. HPLC results showed that rosmarinic acid content in the treated plants with a low concentration of nanocomposite (40 mM) was more affected than plants treated with a high concentration of nanocomposite (60 mM) (26%) and also compared to other treatments. At the molecular level, the result showed that Tyrosine aminotransferase and rosmarinic acid synthase gene expression was positively correlated with both silver nanoparticle concentrations and nanocomposite treatments, but phenylalanine-ammonia lyase gene expression was positively correlated only with nanocomposite at 40 mM concentration.

**Conclude:**

It can conclude that the nanocomposite at low concentration is more likely to induce molecular and biochemical parameters. And also, in the rosmarinic acid biosynthesis pathway, the Tyrosine aminotransferase -derived pathway is more efficient than the phenylalanine-ammonia lyase -derived pathway by causing a nano-elicitor. Therefore, it was concluded that studied elicitor at low concentration, can create plants with higher production capacity.

## Background

Rosmarinic acid (RA) is known as the ester of caffeic acid and 3, 4-dihydroxy phenyl lactic acid. It is an active phenolic compound, found in the Lamiaceae family in lemon balm species *(Melissa officinalis* L.). The RA exhibited various biological activities, including antibacterial, anti-inflammatory, antiviral, antiallergic, antiangiogenic, and antioxidant activities [1]. For some reasons, the pathway of RA biosynthesis has been studied extensively: 1) due to their use in medicine and food 2) their protective activity against microbes. The challenge of elucidating RA biosynthesis, which involves two parallel biosynthesis pathways that must be coordinated, is another reason for the general study of RA. A preliminary report on the RA biosynthesis pathway shows the association of two aromatic amino acids, L-tyrosine (a tyrosine-derived pathway) and L-phenylalanine (a phenylpropanoid pathway) [2]. A coumaroyl-CoA was produced from phenylalanine in three enzymatic steps well-characterized by phenylalanine-ammonia lyase (PAL) catalysis. The second pathway begins with tyrosine, which leads to the production of a 4-hydroxyphenyllactate (4HPL). The first enzyme in this branch is Tyrosine aminotransferase (TAT). The two components of the parallel pathways, 3, 4-dihydroxyphenylacetic acid (DHPL) and caffeine-CoA, are linked together by rosmarinic acid synthase (RAS) to catalyze a trans-sterilization reaction [3]. A research by Kim et al. [4] was shown that methyl jasmonate (MJ) treatment in the *Agastache rugosa* cell culture rapidly increases PAL enzyme activity but decreases TAT enzyme activity in the RA biosynthesis pathway. On the other hand, Yan et al. [5] reported that elicitors increase the RA biosynthesis in the hairy roots of *Salvia miltiorrhiza*, which is related to the tyrosine-derived pathway.

Nanotechnology covered almost all current areas of science such as physics, biology, chemistry and many other fields [6]. Nanotechnology can improve the plant's capacity to absorb the nutrition of agricultural products. In recent years, researchers have used nanoparticles to increase crop quality, plant growth and disease control in agriculture. One of the nanoparticles in nature is graphene (G) which is made of a thick sheet of carbon atoms and is compressed into a matrix [7]. Graphene has a two-dimensional carbon structure with impressive mechanical, chemical, and thermal properties that create different chances for future systems [8]. In recent years, silver nanomaterial has received attention due to their extraordinary chemical properties and improved new technologies. New applications in silver nanomaterials (AgNP) studies include diagnostic imaging, catalysis, therapy, food industry, environmental conservation, anti-microbial and cytogenetic properties, protein-based biosensors finally, effective vehicles for drug delivery [9–11]. A composite material such as nanoparticles / graphene has various properties like chemical, thermal, mechanical, and electronic [12]. Miralles et al. [13] explain the mechanism of environmentally manipulated nanoparticles that support the understanding of the graphene release pathway in an environmental remediation program as agricultural delivery systems and accidental release. By controlling the chemical composition, size, and shape of materials at the nanoscale, the nanomaterials' properties can be changed. Currently, with functional groups, such as conjugation with plant products, nanomaterials can be modified to introduce a wide range of future biotechnology applications [14]. To avoid the accumulation of graphene with a better specific distribution, the metals are placed in graphene layers. Metal nanoparticles can be integrated into graphene sheets to prevent dry graphene accumulation. To access both graphene sheets surfaces of, metal nanoparticles increase the distance between the graphene sheets as spacer [15]. Nanoparticle / Graphene composites have biological and agricultural applications [16].

Plant extracts are potential source of reducing agents for the synthesis of metal nanoparticles such as silver nanoparticles [17]. Recently, researchers have recorded a simple and an eco-friendly system for synthesizing graphene using plant extracts [18]. Among the plants studied, *M. officinalis* was selected for the synthesis of graphene with stable dispersion. Elicitors such as nanomaterial can positively affect the synthesis of secondary metabolites in medicinal plant species [19–21]. Some changes in plant growth were detected after treatment with nanoparticles. Nanomaterials can enhance changes in molecules and cellular structures. The chemical composition of nanoparticles plays a crusial role in their toxicity and the stress caused by the size, surface, and shape of particles [8]. Also, toxic nanoparticles increase the production of reactive oxygen species by changing the shape of cell membranes, and changing the permeability of nanoparticles.The stresses induced by nanomaterial results in the production of secondary metabolites [22].

So far, only a few types of research have been conducted on nanocomposites in plants to evaluate plant production. Hence, it has considered biologically synthesized silver nanoparticle, graphene, and AgNP/ graphene nanocomposite on some genes involved in the rosmarinic acid biosynthesis pathway and then phytochemical changes of rosmarinic acid in *M.officianalis.*

## Methods

### Material and treatments

#### Growth conditions of plant

The study was performed in a randomized factorial design in the Sana Institute of Higher Education. Seedlings of *M. officinalis* were purchased (Seed and Plant Improvement Institute, Karaj, Iran.), and planted in soil. After the appearance of 3 leaves of the plant (after three weeks), treatment was done with the foliar application (2 times in 3 weeks). Treatments included 1: green synthesis of silver nanoparticles (600 ml) (synthesized using silver nitrate and ethanolic extraction according to the following method) (in different concentrations 0, 40, 60 mM), 2: graphene, and 3: AgNP / graphene nanocomposite. Greenhouse conditions for plant growth were 16 h of light (PPFD in µmol m-2 s-1 or W m-2 light), and 8 h of darkness, relative humidity of 60%, and an average temperature of 5 ± 25 °C. This experiment was performed with three replications in each treatment (three biological repeats and three technical repeats). Plant treatment was repeated three times every week. After two weeks, the plant was gathered for molecular and biochemical analysis.

### Preparation of extracts

For the extraction of *M. officinalis* leaves, the ethanolic extraction method was performed. The solvent was a mixture of ethyl alcohol and water and ethyl alcohol (in a 1:1 ratio). *M. officinalis* plant leave (5 g) was added into an ethanolic solvent (25 ml) and was pounded for 15 min. The mashed was remixed with an extra solvent (80 ml). The extraction process was performed in darkness, by a mechanical stirrer with a rotational speed of 750 rpm for three hours. The extracted solution was filtered twice through the Whatman filter paper No 1. The extracts were stored in a dark tube at 4 °C [23].

### Preparation of metal nanoparticles (AgNP) suspension

Typically, plant extract (10 mL) was added to 1 mmol L^−1^aqueous AgNO_3_ solution (90 mL) for the Ag^+^ reduction. The solution was stirred for two h at 60^∘^C. The synthesized nanoparticles were centrifuged at 15 000 rpm for 20 min for purification (twice), followed by pellet re-dispersion in deionized water. The purified powder of nanoparticles was used for further analyses.

### Synthesis of Graphene

For the synthesis of graphene, plant extracts (50 g/L) was kept in low power sonication of expanded graphite (Samjung C & G, Korea, 1–10 g/L) for 24 h by controlling the temperature lower than 30^0^C with a continuous flow of water in the ultra-sonication bath (JACUltrasonic4020P). After sonication, the dispersion was left overnight to separate large unstable graphite aggregates. To maintain the dispersions of graphene, stabilized in water by the sodium cholate surfactant, at concentrations of 0.3 mg/mL. Then deionized water (46 mL) was added to this paste, and the solution was evaporated at 95 °C for 40 min. The graphene solution was centrifuged at 1500 rpm for 90 min to obtain a stable dispersed graphene solution. For removal of sulfate ions, the final solution was washed with distilled water. Then solution was dried in an air-oven at 50 °C for 2 d. The final powder was used for the next step of the analysis.

### Preparation of nanocomposites suspension

Ag/Graphene nanocomposites were prepared by reducing graphene and silver ions together. First, expanded graphite (1 g) was added to AgNP solution (100 mL) and was mixed in deionized water (50 mL). The reaction mixture was sonicated in the ultra-sonication bath (JAC Ultrasonic 4020P) for one day at 60^∘^C. Then, hydrazine hydrate (10 mL) was then gradually added to the mixture by magnetic stirring. The sonication temperature was kept by controlling the water volume in the bath and covering it with a lid. The temperature of the mixture was increased and kept at 95 °C for one h. Finally, the obtained powders were washed several times with deionized water and ethanol. After centrifugation, the prepared samples were dried in an air-oven at 50 °C for 48 h.

### Characterization of nanocomposites

UV–visible spectra of nanocomposites were measured by using a UV–visible spectrophotometer (UV-1601, Shimadzu, Japan). After freezing of the nanocomposites solution, the composition and structure were investigated by field emission SEM (Carl Zeiss, LEO-1530). X-ray diffraction analysis was done for analysis of AgNP and graphene (Bruker AXS, D8 Discover with GADDS). Nano Raman spectroscope (NT-MDT, NTEGRA) was used for discussing about the structural characters of graphene sheets (single-layer or multilayer property) of the sheets.

### Biochemical assay

### Total carbohydrate determination

Total carbohydrates content was measured using enthrone modified method [24]. Plant ethanol extracts were evaporated at 70 °C under a vacuum. For removal of chlorophyll, chloroform was mixed with plant extracts and then centrifuged for 5 min. Enthrone solution was prepared with the mixture of enthrone (150 mg) in H_2_0_2_ (30 ml) and H_2_SO_4_ (76 ml)_._ Extract (1 ml) was incubated with prepared enthrone solution at 90 °C for 10 min. The absorbance of final solution was read at 630 nm.

### Total protein assay

Total protein content was measured by the Bradford method [25]. The standard solution used was Bovine serum albumin (BSA), and the absorbance of total protein content was read by a spectrophotometer at 550 nm.

### Determination of total phenol and flavonoid content

The total phenolic using Folin–Ciocalteu and flavonoid content using aluminum chloride methods were measured, respectively. Total phenolic was analyzed based on mg of gallic acid equivalents (GAE) per g of DE. Flavonoid content was analyzed based on mg of quercetin equivalents (QE) per g of DE. Details of methods have been previously reported [26].

### Extraction and HPLC analysis for determination of RA content

Dried weighted plant (0.1 g) was powdered and then extracted using methanol-H_2_O (7:3) (10 ml) by an ultra-sonication for 30 min. The centrifuged solution with 70% methanol was reached to volume and then was filtered through a syringe filter (Liu et al. 2013). Filtered solution (10 μl) was injected into the HPLC apparatus for further analysis. For determination of RA content in the plant extract, an HPLC apparatus with the following instrument was used: C18 column with dimensions of 250 × 4.6 mm, particles: 5 μm. In the separation section, a gradient elution in a linear mode was done by the proportion of acetonitrile to acetic acid (2.5% v/v). Detector of HPLC was a photodiode array. RA content was measured by retention time in the curve, and standards compound [27, 28].

### Measurement of gene expression level by extraction of total RNA, cDNA synthesis and Real-time analysis

cDNA was used as a template for PCR real-time analysis. Primers were designed by Primer 3 online software and OLIGO5 analyzer software (Table 1). PCR was done using the SYBR® Green with Taq DNA enzyme polymerase (Thermo Scientific) in a BIO-RAD real-time PCR machine according to the manufacturer's recommendations. Actin as a housekeeping gene for normalization was used. For data analysis, a comparative gene expression method (2-^ΔΔcT^) was used [29, 30].

**Table 1:** Designed primer sequences in this study

Gene name	Primer sequence (5**′**–3**′**)	Product length	Tm
*β*-*Actin*	Actin-f TGTATGTTG CCATCCAGG CCG Actin-r AGCATGGGGAAGCGCATAACC	0.996	55
*RAS*	RAS-f ACGCCCCGACCTCAACCTTATC RAS-r AAGTGGTGCTCGTTTGCCACG	0.991	53/4
*PAL1*	PAL-f GCCGAAGTCATGAACGGAAAGC PAL-r CGCAGCCTTAACATAACCGCTC	0.996	54/2
*TAT*	TAT-f CCTACAAGCTACCAGCCGACTC TAT-r AGCCCGTAGATTGGGAAACACG	0.993	54/4

### Statistical analysis

The data were organized as factorial, completely randomized design (CRD). Data analysis was done by one-way ANOVA and significant difference at the 5% level (*P* ≤ 0.05) in the SAS software (version 9.1.3, SAS Institute, Cary, NC).. Means differences among data were performed by least significant difference (LSD) methods and the graphs were drawn in Microsoft Office Excel 2010.

## Results

### SEM images of synthesized AgNP / graphene nanocomposite on *M.officianalis* leaves

By SEM images, the size and shape of synthetic silver nanoparticles were shown.

**Fig. 1:**
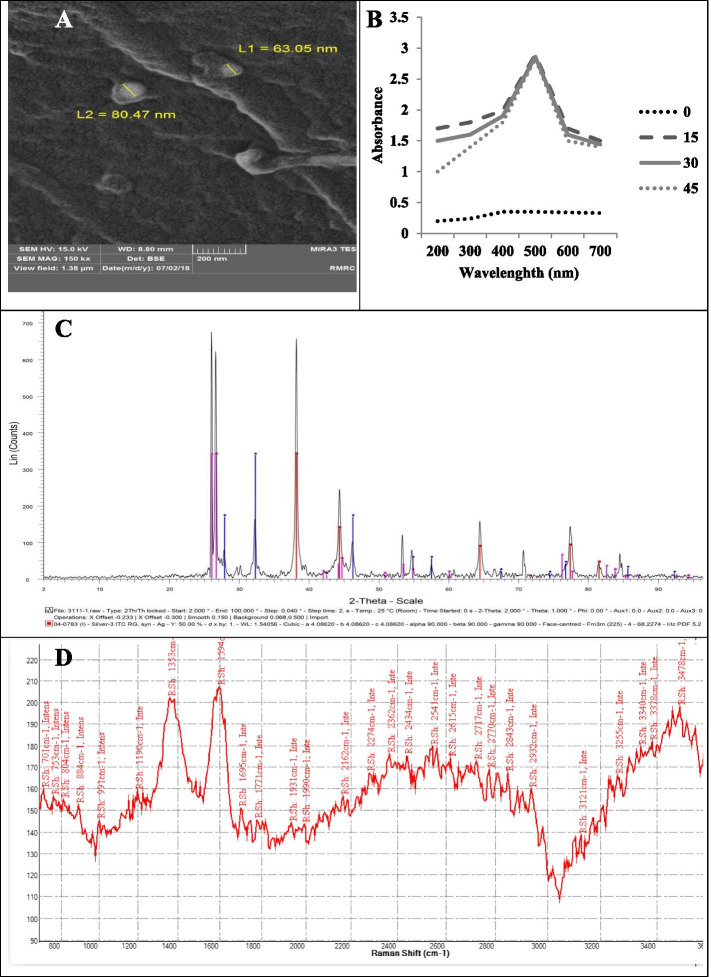
Characteristic of synthesized AgNP and Graphene with *M. officianalis* leaves, **A:** Image of synthesized AgNP/graphene nanocomposite by SEM microscope, **B:** UV–visible spectrum of biosynthesized AgNPs exhibited a peak at 480 nm, **C:** XRD spectra of graphene, and AgNPD: Raman spectra of graphene

The results showed that AgNP has a spherical shape with an average diameter of about 50–80 nm (Fig. 1 a). The absorption range of *M. officianalis* extract from 300 to 700 nm at different wavelengths was showed a peak at 480 nm absorption bands in UV–vis spectra (Fig. 1 b). XRD analysis showed distinct diffraction peaks at 38.1°, which indexed the planes (225), of the cubic face-centered silver nanoparticle. XRD data indicated that AgNP were successfully prepared from *M. officianalis* plant extract (Fig. 1 c). Raman spectroscopy was used to detect the crystalline structure of graphene (Fig. 1 d). For plant extract samples, two peaks at about 1353 and 1594 cm − 1 was assigned to the D and G-band. The D- and G-band band is related to the vibration of sp^3^ and sp^2^ carbon atoms of disordered graphene nanosheets. It was recognized that the G and 2D bands of peak showed monolayer graphene sheets,

### Biochemical assay result

**Fig. 2:**
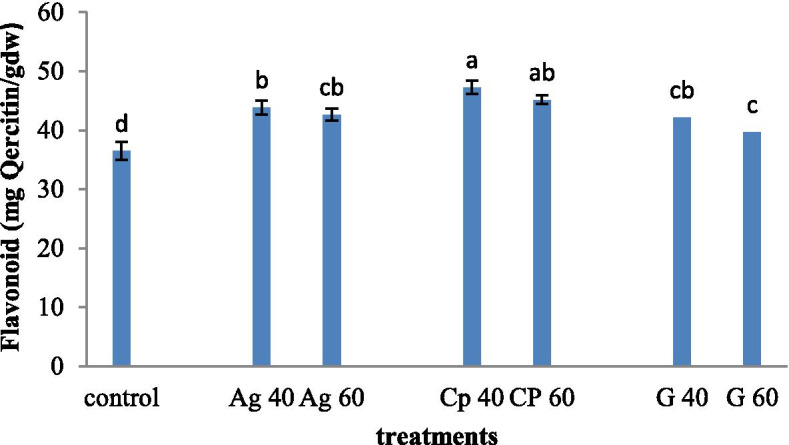
Treatment of synthesized graphene nanoparticles (G40 and G60), silver nanoparticles (Ag40 and Ag60), and nanocomposites (CP40 and CP60) on flavonoid level in *M. officinalis* plant. The different letters at the top of the graphs indicate the significance of the results at the 5% level

The flavonoid analysis results showed the highest range of flavonoid in the plant treated with synthesized nanocomposites at both 40 and 60 mM concentrations (46 mg quercetin. dry weight). The lowest content of flavonoid was observed in the control plant (36 mg quercetin per gram dry weight). The flavonoid analysis results showed no difference between plants treated with AgNP at both 40 and 60 mM concentrations (Fig. 2).

**Fig. 3:**
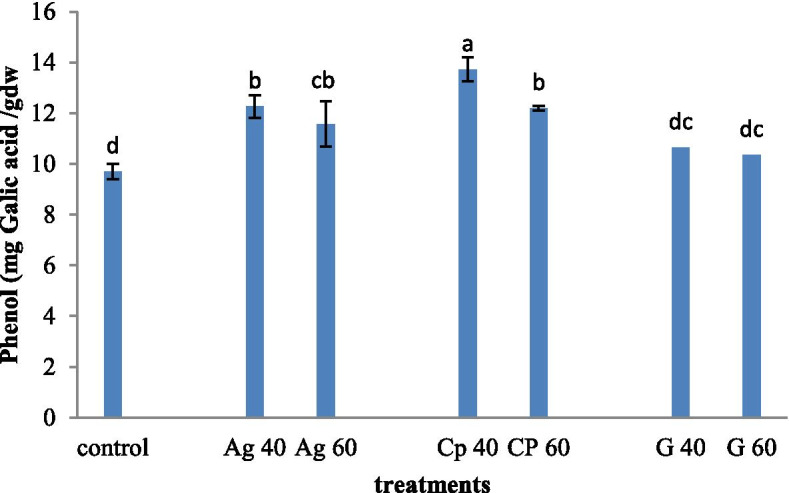
Treatment of synthesized graphene nanoparticles (G40 and G60), silver nanoparticles (Ag40 and Ag60), and nanocomposites (CP40 and CP60) on phenol content in *M. officinalis* plant. The different letters at the top of the graphs indicate the significance of the results at the 5% level

Based on the result, the highest content of phenol (13 mg Gallic acid / g dry weight) was observed in the nanocomposite treated plant at a concentration of 40 mM. While in the control plant sample and graphene treated plant at both concentrations of 40 and 60 mM, the lowest phenol content was observed (9.45 mg gallic acid / g dry weight). No significant differences were observed in plants treated with concentrations of 40 mM silver nanoparticles and 60 mM nanocomposites (Fig. 3).

**Fig. 4:**
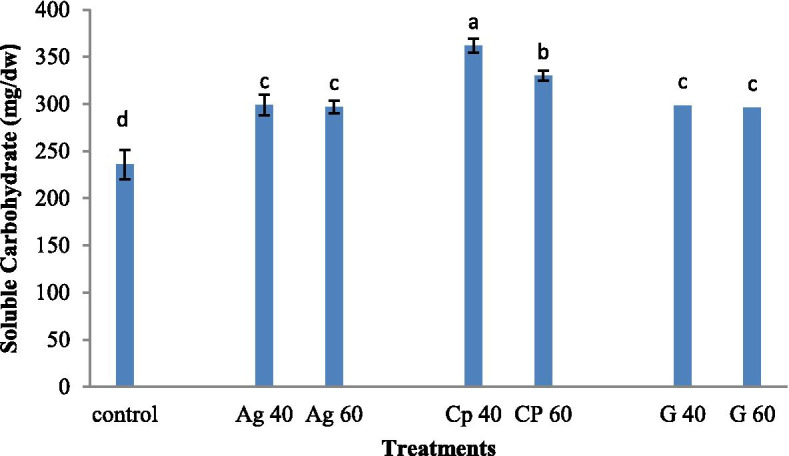
Treatment of synthesized graphene nanoparticles (G40 and G60), silver nanoparticles (Ag40 and Ag60), and nanocomposites (CP40 and CP60) on total carbohydrate in *M. officinalis* plant. The different letters at the top of the graphs indicate the significance of the results at the 5% level

The highest content of soluble carbohydrate (368 mg / g dry weight) was recorded in plants treated with AgNP/graphene nanocomposites and AgNP at a concentration of 40 mM. The control sample showed the lowest content of soluble carbohydrate (230 mg / g dry weight). No significant difference in the soluble carbohydrate content was observed between the treatments of silver nanoparticles and graphene at concentrations of 40 and 60 mM (Fig. 4).

**Fig. 5:**
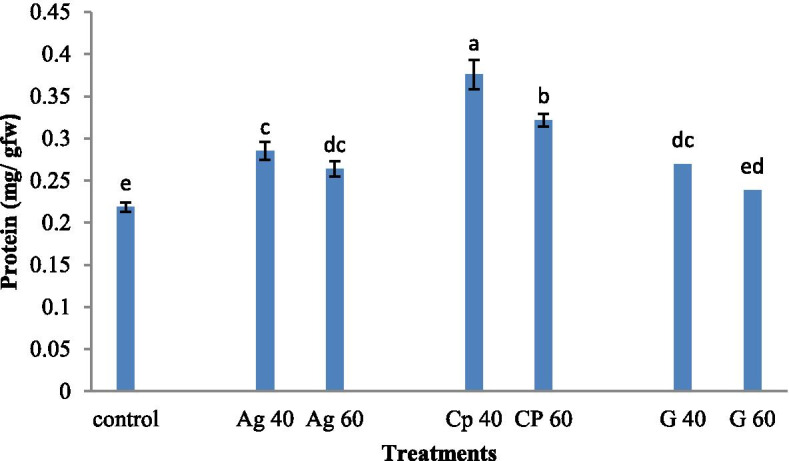
Treatment of synthesized graphene nanoparticles (G40 and G60), silver nanoparticles (Ag40 and Ag60), and nanocomposites (CP40 and CP60) on flavonoid level in *M. officinalis* plant. The different letters at the top of the graphs indicate the significance of the results at the 5% level

Nanocomposite treated plants with a concentration of 40 mM showed the highest content of total protein (0.37 mg/g dry weight).Plants treated with silver nanoparticles at concentrations of 40 and 60 mM showed almost the same content of total protein (0.28 mg/g dry weight). Plants treated at both 40 and 60 mM concentrations of graphene, and control samples showed lower total protein content (0.29 mg / g dry weight). There was no significant difference in total protein content between AgNP treated plant at concentrations of 40 and 60 mM and the nanocomposites treated plant at a concentration of 60 mM (Fig. 5).

**Fig. 6:**
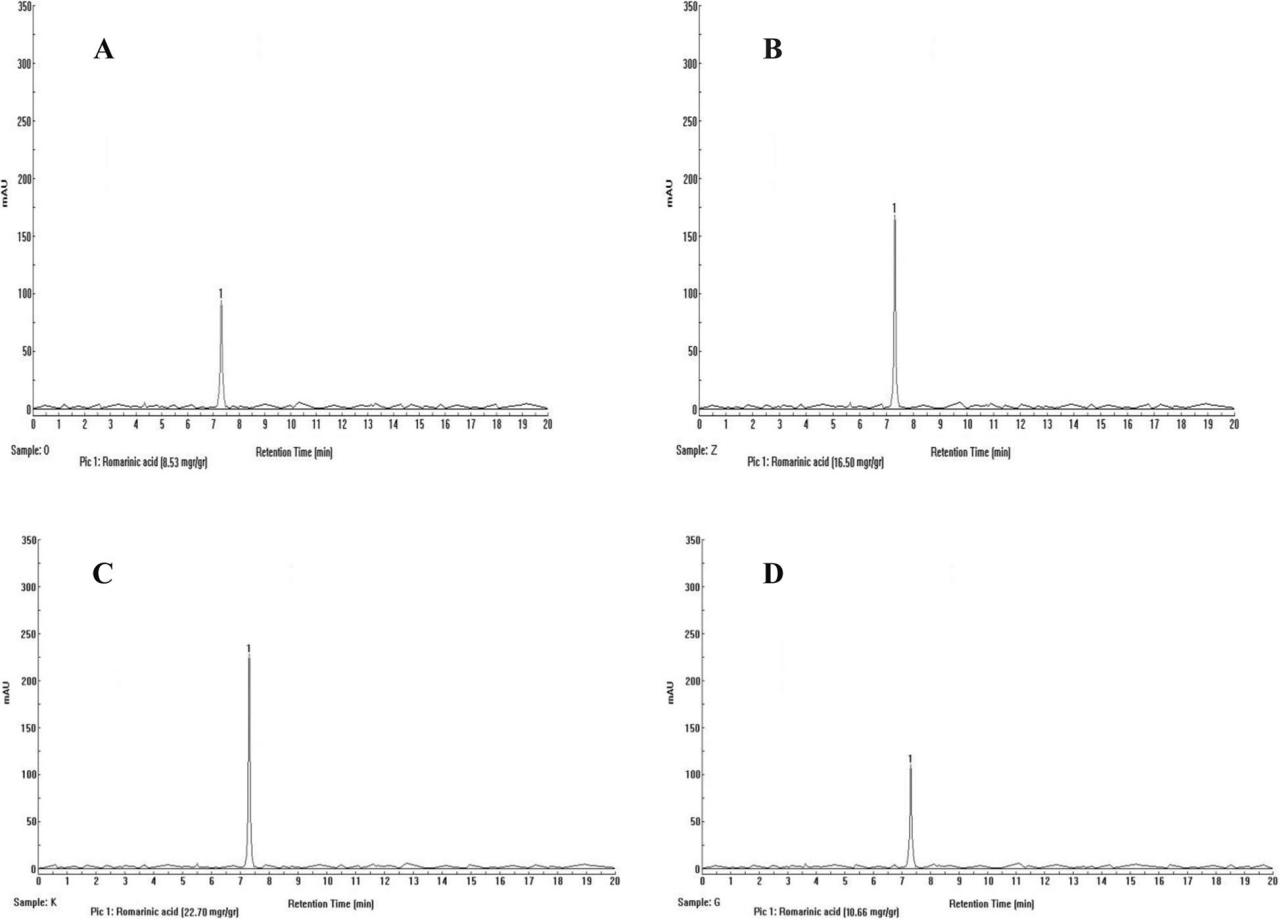
Chromatograms of concomitant treatments at a concentration of 60 mM **A:** control **B:** AgNP **C:** nanocomposite **D:** Graphene on rosmarinic acid level of *M. officianalis*

HPLC analysis of *M. officinalis* extracts recorded a major peak according to valid standards, as rosmarinic acid. The retention time of RA was 7.5 ± 0.4 min (Fig. 6).

**Fig. 7:**
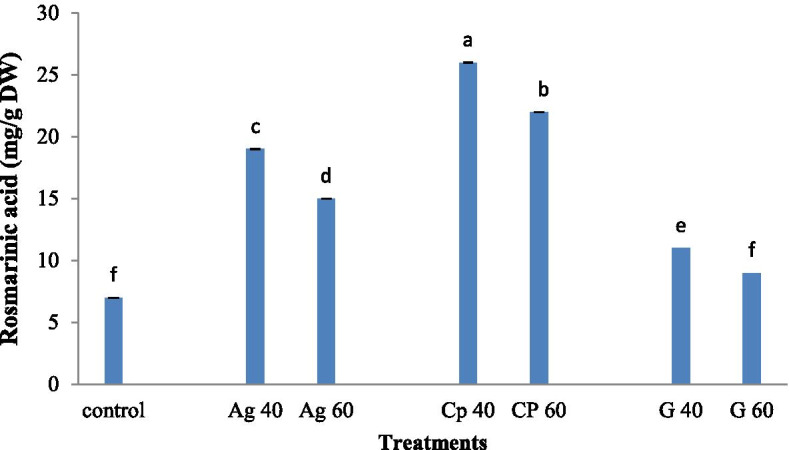
Treatment of synthesized graphene nanoparticles (G40 and G60), silver nanoparticles (Ag40 and Ag60), and nanocomposites (CP40 and CP60) on RA content in *M. officinalis* plant. The different letters at the top of the graphs indicate the significance of the results at the 5% level

Comparison means of a plant treated with different concentrations of AgNP, graphene, and AgNP / graphene nanocomposites on RA content in *M. officinalis* plant is shown in Fig. 6. The highest RA content was observed in nanocomposite treated plants at a concentration of 40 mM (**26** mg/g DW), and the lowest RA content was recorded in the control sample and 40 mM graphene treated plant (7 ± 4 mg/g DW). A comparison between all treatments showed that the concentrations of 40 mM studied nano-elicitor produced higher content of rosmarinic acid. Plants treated with graphene at 60 mM and the control sample didn’t show a significant difference in the content of rosmarinic acid (Fig. 7).

### Results of gene expression

**Fig. 8:**
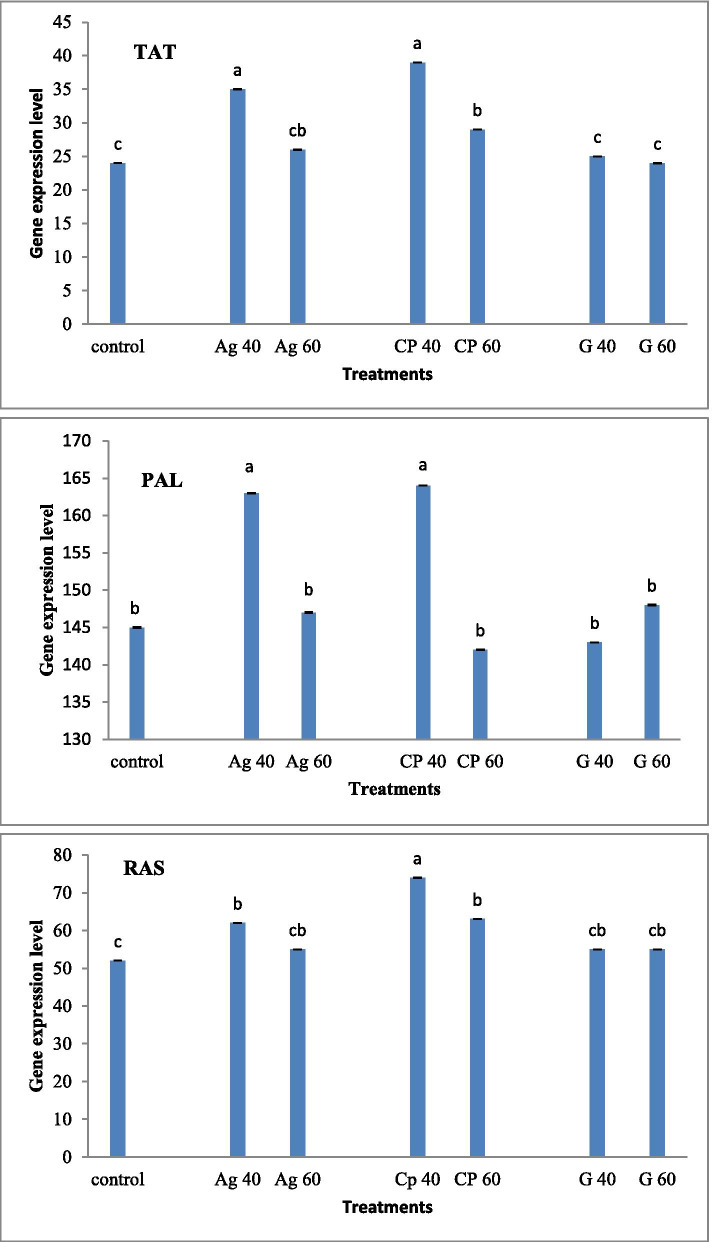
Treatment of synthesized graphene nanoparticles (G40 and G60), silver nanoparticles (Ag40 and Ag60), and nanocomposites (CP40 and CP60) on gene expression of TAT, RAS, PAL of *Melissa officinalis*. The different letters at the top of the graphs indicate the significance of the results at the 5% level

The effect of exogenously synthesized nanoparticles on the induction of TAT, RAS and PAL gene expression was investigated by real-time PCR analysis (Fig. 8). In the analysis of gene expression, a significant increase in TAT expression was observed in plants treated with nanocomposite, and AgNP at concentration of 40 and 60 mM compared to the control sample. The highest expression of TAT gene was observed in plants treated with AgNP, and nanocomposites at a concentration of 40 mM. There is no significant difference between graphene treated plants and control sample. For the RAS gene, both exogenous AgNP and nanocomposite at a concentration of 40 mM positively induced RAS expression, with the highest expression at 40 mg/l nanocomposites treated plant compared to the control plant sample. Significant induction of PAL gene expression (74.17) was observed only in plants treated with 40 mg/l nanocomposites and AgNP. There was no difference between plants treated with both graphene concentrations and plants treated with 60 mM AgNP, and nanocomposite compared to the control sample.

## Discussion

Several types of research have been done on the manipulating of RA production to increase the hydroxylated phenolic compounds. In some studies, the mechanism of different elicitors, including salicylic acid, methyl jasmonate, yeast elicitor, abscisic acid, chitosan, and silver ion on the phenolic compounds, especially the production of RA, has been investigated [31]. While many studies have investigated the plants' responses elicitors, the mechanism of increased rosmarinic acid production under AgNP/ graphene nanocomposite treatment has not yet been studied.

Various researchers have observed the contradictory effects of AgNP on plants. The response of plant cells to the nanoparticle causes changes in gene expression, related biological pathway, and finally leads to plant growth and development [32, 33].

Recently, simple and eco-friendly strategies have been used to produce metal/graphene nanocomposites using plant extracts [17, 18]. Jing et al. [34] reported that graphene/gold and graphene/silver nanocomposites using *Xanthium strumarium* leaves have less toxic, biocompatible and suitable biological applications. The present study investigated the effect of green synthesis of AgNP, graphene, and AgNP / graphene nanocomposites (at concentrations of 40, and 60 mM) on biochemical, phytochemical, and some genes involved in the RA biosynthesis pathway in *M.officinalis* plant. Overall, the results of the present study showed the possible effects of silver nanoparticles, graphene and silver/graphene nanocomposites on the cellular pathways related to RA production in *M. officianalis* plants. Also, elicitor concentration plays an important role in stimulating biochemical and molecular processes in *M. officinalis* plants.

In this study, plants treated with nanoparticles and nanocomposite (at a concentration of 40 mM) had a positive effect on the total phenol content of *M. officianalis*. Some studies have shown that the use of nanoparticles in plants increases phenol production [35, 36]. For example, artemisinin [37] and diozengine content [38] as essential drugs, in the plants treated with nanoparticles have increased. The concentration of phenolic compound after treatment with titanium nanoparticle increased by 22% in the extracellular culture of *Arthrospira platensis* and *Haematococcus pluvialis*, respectively [36]. In one study, a significant 39% increase in the artemisin concentration was reported after AgNP treatment in *Artemisia annua* root. This increase was related to H_2_O_2_ production, CAT activity, and lipid peroxidation [37]. *Trigonella foenum*-*graecum* L. exposed to silver nanoparticles showed a high plant growth rate, and diagenetic concentration [37]. Isovitoxin and ferulic acid increased in barley after nanoparticle treatment [35, 38]. Fe and Ag nanoparticles have a positive effect on seed germination and bioavailability in barely [39].

Mean comparison of AgNP, graphene and nanocomposits treatments showed that nanocomposites treatments were more effective on the RA level than plants treated with graphene, and AgNP alone. AgNP, graphene, and nanocomposites treatments showed concentration-dependent behavior on the *M. officinalis* plant. The synthesis of silver nanoparticles by reducing of silver nitrate (AgNO_3_) during treatment with *M. officinalis* extract showed a yellow to light-brown reaction. In research by Nokandeh et al. [40], a color change was observed after extraction of *stevia rebaudioside*, which showed the synthesis of AgNP. In the present study, a maximum wavelength of 480 nm was observed, due to the stimulation of the plasmon absorption spectrum of the extract, which leads to discoloration. [41]. Surface plasmon resonance (SPR) bands are related to the size, shape, morphology, composition, and environment around the synthesized nanoparticles [42]. SEM microscopy image reported spherical shape and 23.7 nm in size of synthetic AgNP. According to the results, the interaction between the reductions of biological agents such as terpenoids, alkaloids, flavonoids, and metal atoms determined the size and morphology of AgNP [43].

Based on these results, the use of AgNP, graphene, and silver / graphene nanocomposites in *M. officinalis* leads to an increase in the content of soluble carbohydrate, protein, total phenol, and flavonoid. This result also depends on the concentration of treatments, which leads to increase protein synthesis and induction of carbohydrates. AgNP improved growth due to the increase in soluble carbohydrates [44]. In this study, the *M. officinalis* plant treated with 40 mM nanocomposite increased the soluble carbohydrates content. Each of these nanoparticles activates a signal pathway for the production of soluble carbohydrate and, when combined, activates more signal pathways for the production of soluble sugar. The concentration of flavonoids increased in nanocomposite treated plants. An increase in the end-products of the phenylpropanoid pathway such as flavonoids, phenol, and anthocyanin concentration in stress-treated plants are essential for the stabilizing effects of elicitors [45]. AgNP can regulate key genes involved in the biosynthetic pathways of flavonoids and anthocyanin in *A. thaliana* [36, 46]. AgNP has increased biochemical parameters, including carbohydrate and protein contents, chlorophyll, antioxidant enzymes in mustard, beans, and maize [47]. In the current study, nanocomposite treatment at a concentration of 40 mM resulted in induction in total protein content. Also, nanocomposite at concentration of 60 mM had a more positive effect on biochemical properties such as total protein and carbohydrate compared to other treatments. On the other hand, graphene treatment was more affective on the protein level, thus; different nanoparticles can regulate different pathways of enzyme activity.

In a study, SiO_2_NP increased photosynthesis, and improved carbonic anhydrase enzyme activity, followed by the synthesis of the photosynthetic pigments. As a result, nanoparticles increase gas exchanges and chlorophyll fluorescence, resulting in the efficient production of some phytochemicals, photosynthesis (PSII), transpiration, stomata adsorption and electron transfer rate [48]. After plant treatment with nanoparticles, photosynthesis rate increased in carbohydrate and nutrients content as the final product of photosynthesis [49]. Also, treatment of the plant with nanoparticles increases the absorption of iron, calcium, and water, thereby increasing plant growth factors. The results showed that plant cells treated with nanoparticles are involved in multiple pathways that ultimately affect plant growth and plant production [50, 51].

Stress may be an important cause of protein production. Also, the increase in protein level in plants under stress is probably due to the required energy for the mechanisms involved under stress conditions [52].

Graphene has a phototoxic effect on plant cells due to its high aggregation. This effect of graphene leads to cell death and concentration-dependent behavior. Many observations have shown that graphene can be transported regularly to plants (between cells and in different plant organs) and leads to strong intercellular interactions that cause fundamental changes in the gene expression, which eventually exert its toxic effect [53]. Akhavan [53], in his research, showed Size-dependent genotoxicity of graphene nanoplatelets in human mesenchymal stem cells.

The high production of plant compounds caused by graphene induction inhibited plant growth and reduced plant yield, due to the toxic effects of nanomaterials [54]. Graphene can be transferred from roots to plant stems and eventually to cytoplasm and chloroplasts [55]. It has a delaying effect on plant germination, reduced plant length, root diameter, roots number, and dry weight [56]. Many factors effect on the graphene function, such as the chemical and physical properties, concentration and biological effects of the nanomaterial, shape, size, solubility, composition, and aggregation. Among these factors, surface and size are essential features in terms of plant toxicity. A high surface to volume ratio increases absorption and interaction, leading to different cells' biological activity [57].

Because photosynthesis is essential for plant growth, graphene significantly inhibits chlorophyll production and acts as a secondary surface on the plant leaves, reducing plant growth and production. The mechanism of phototoxicity of graphene includes mechanical damage and shading effects that lead to light scattering on the plant surface and consequently reduce photosynthesis rate [58]. The anchoring of metallic nanoparticles to the nano-layers of graphene helps prevent the accumulation of graphene sheets and high dispersion in dry condition. Metal nanoparticles increase the distance between the graphene sheets, and then make both graphene sheets available [59].

In the present study, the plants treatment with 40, 60 mM nanocomposite increased the phenol and RA production in *M. officinalis*. As shown in Fig. 8, AgNP and nanocomposite treatment at all concentrations significantly up-regulated the expression of TAT and RAS genes. But AgNP and nanocomposite treatment only increased PAL gene expression only at 40 mM concentration. In the current study, a positive relationship was observed between PAL enzyme activity, phenol, and flavonoid accumulation. Because the PAL enzyme plays an essential role in the biosynthesis of total phenol content via the phenylpropanoid pathway, the molecular regulation in the biosynthesis of phenolic content associated with the PAL gene expression. Multi-gene families encoded different isoforms of PAL in many plants [60]. Different isoforms are expressed in a specific tissue. Every modification of them causes a change in PAL enzyme activity, which induces phenylpropanoid pathway and end-products. Research was done by Gayoso et al. [61] showed that among six PAL genes expressed in tomato plant roots, PAL2 showed only the highest expression level, followed by PAL3, PAL4, and PAL6. It appears that the potential effect of nanocomposite and AgNP on the expression of PAL1 gene level dependent on the concentrations of nano-elicitor; It is possible that low concentrations of nanocomposite and AgNP (40 mM) treatment may induce the expression of other isoforms of PAL gene in *M. officinalis* plant. Increased RA production and expression of the main genes of the RA biosynthesis pathway (RAS and PAL1, TAT) by nano-elicitors have been reported slightly in plants.

One of medicinal effect of graphene was genotoxicity that was approved in different researches in human mesenchymal stem cells, anti-apoptotic genes transcripts patterns, and spermatozoa and apoptotic of graphene in mice [62–64].

In research was done by Banchi and their collaborations [65], it was showed that graphene-based materials do not impair physiology, gene expression (HSP70-1 gene) and growth dynamics (photosynthetic pigment contents) of the aeroterrestrial microalga *Trebouxia gelatinosa*.

Increased RA production level after *M. officinalis* treatment at 40, and 60 mM concentration of AgNP and nanocompoite can be due to the induction effect of nano-elicitors on the RAS and TAT gene expression level. The results of various researches have shown that there are one or two branches for the RA biosynthesis pathway. Some of them indicated that TAT activity (not PAL) is related to the biosynthesis pathway of RA and phenolic compounds [66].On the other hand, the others studies showed the involvement of PAL in the biosynthesis pathway [4, 61]. The TAT activity was thought to be involved in RA production. For example, Lu et al. [67] and Kim et al. [4] focused on the role of TAT gene in the RA biosynthesis pathway in *Perilla frutescens* and *Scutellaria baicalensis* (species of Lamiaceae family), respectively. In a study by Li et al. [68], the importance of PAL-derived pathway than TAT-derived pathway in the RA accumulation in *Dracocephalum tanguticum* plant was reported. The resent study; showed that the TAT gene expression was positively correlated with the RA accumulation, highlighting the importance of regulation in the RA biosynthesis pathway. The present study results indicated that in the comparison between the TAT-derived pathway and the PAL-derived pathway, the first pathway to induce biosynthesis of RA by nano-elicitor in the *M. Officinalis* plant is more probable.

## Conclusion

In the present study, graphene/metal nanocomposites were synthesized from the graphite by mild sonication method with the plant extracts of *M. Officialanis*. Plant treated with synthesized AgNP/graphene nanocomposites showed higher biochemical parameters, RA content, and TAT, RAS and PAL gene expression, than individual nanoparticles. In the present study, it was observed that nanocomposites due to the performance of two nanoparticles could have more potential effect on phytochemical and molecular parameters. The results showed that nano-elicitor (AgNP and nanocomposite) effected on the expression of PAL gene at low concentration (40 mM) but induced RAS and TAT gene expression at both concentrations of nano-elicitors. Therefore, it can be concluded that the application of nano-elicitor in *M. officialanis* has no effect on the phenylpropanoid pathway, and this probably leads to more RA production in the tyrosine-derived pathway. Compared to the control, the studied parameters in *M. officialanis* treated plant with different concentrations of graphene were lower than AgNP or nanocomposite. It was suggested that graphene showed specific inhibitory effects on the production of the *M. officilanis* plant. This study has introduced the optimum concentration of synthesized nanoparticles in the field of agriculture and pharmaceutical.

## Data Availability

Al data generated or analyzed during this study are included in this published article.

## Abbreviations

AgNP: Ag nanoparticle
ABA: Abscisic acid
BSA: Bovine serum albumin
IAA: Indole-3-acetic acid
G: Graphene
Silver (Ag): Nanoparticle
*M. officinalis*: *Melissa officinalis*
LSD: Least significant difference
CRD: Completely randomized design
RA: Rosmarinic acid
JAL: Jacalin-related lectin
CRD: Completely randomized design
LSD: Least significant difference
PAL: Phenylalanine-ammonia lyase
4HPL: 4-Hydroxyphenyllactate
DHPL: 3, 4-Dihydroxyphenylacetic acid
RAS: Rosmarinic acid synthase
MJ: Methyl jasmonate
